# Sustainable Bio-Gelatin Fiber-Reinforced Composites with Ionic Coordination: Mechanical and Thermal Properties

**DOI:** 10.3390/ma18194584

**Published:** 2025-10-02

**Authors:** Binrong Zhu, Qiancheng Wang, Yang Wei, Jinlong Pan, Huzi Ye

**Affiliations:** 1 Jiangsu Carbon Sequestration Materials and Structural Technology of Bamboo & Wood Research Center, College of Civil Engineering, Nanjing Forestry University, Nanjing 210037, China; zbr2022@njfu.edu.cn (B.Z.); wqc13611511230@163.com (Q.W.); wy78@njfu.edu.cn (Y.W.); 2 Key Laboratory of Concrete and Prestressed Concrete Structures of Ministry of Education, Southeast University, Nanjing 211189, China

**Keywords:** bio-gelatin composites, fiber reinforcement, impact behavior, thermal performance, ionic coordination

## Abstract

A novel bio-gelatin fiber-reinforced composite (BFRC) was first developed by incorporating industrial bone glue/gelatin as the matrix, magnesium oxide (MgO) as an additive, and natural or synthetic fibers as reinforcement. Systematic tests evaluated mechanical, impact, and thermal performance, alongside microstructural mechanisms. Results showed that polyethylene (PE) fiber-reinforced composites achieved a tensile strength of 3.40 MPa and tensile strain of 10.77%, with notable improvements in compressive and flexural strength. PE-based composites also showed excellent impact energy absorption, while bamboo fiber-reinforced composites exhibited higher thermal conductivity. Microstructural analysis revealed that coordination between Mg^2+^ ions and amino acids in gelatin formed a stable cross-linked network, densifying the matrix and improving structural integrity. A multi-criteria evaluation using the TOPSIS model identified the BC-PE formulation as the most balanced system, combining strength, toughness, and thermal regulation. These findings demonstrate that ionic coordination and fiber reinforcement can overcome inherent weaknesses of gelatin matrices, offering a sustainable pathway for building insulation and cushioning packaging applications.

## 1. Introduction

With the increasing depletion of global non-renewable energy resources, growing research efforts have focused on the development and utilization of renewable energy and green building materials. Among these, concrete as the second most consumed material globally after water, with an annual consumption exceeding 30 billion tons, imposes a significant environmental burden [1,2]. Biomass materials have garnered increasing attention in the construction sector due to their sustainability and multifunctionality. Smirnova et al. [3] developed high-strength bio-concrete through microbially induced calcium carbonate precipitation (MICP), achieving a compressive strength of 52.5 MPa and a cementation depth of 140 mm, demonstrating its potential as a cement substitute. Lee and Choi [4] investigated the performance of mycelium-based composites (MBC) with different matrices in adsorbing atmospheric particulate matter (PM), finding that they outperformed conventional building stones and proving their value in environmental purification applications. Additionally, MBC exhibits versatility across various industrial fields, including construction, with applications ranging from insulation to furniture [5]. Al-Sabaeei et al. [6] explored the potential of crude palm oil (CPO) and its by-products in green building materials, investigating their substitutability, sustainability, and challenges in asphalt and concrete, and demonstrating their feasibility as renewable, eco-friendly, and cost-effective alternatives. The application of biomass materials in the construction sector has thus become a focal point of current research.

Gelatin, derived from animal bones or food waste, has emerged as a promising bio-based polymer binder in construction due to its biocompatibility, eco-friendliness, and mechanical hardening properties. Studies have demonstrated that gelatin, when used as an organic binder, can significantly enhance the hardened mechanical properties, toughness, and durability of composites [7,8]. For instance, Hematibahar et al. [9] investigated the effects of incorporating gelatin powder, apricot shells, and recycled aggregates into conventional concrete, revealing that gelatin powder markedly improved its mechanical strength, impact toughness, and durability. In another study, a novel thermoplastic composite was developed by entirely replacing cementitious materials with mammalian gelatin solution. Three-dimensional printing experiments further demonstrated its ability to achieve up to 80° overhang printing, underscoring its potential for free-form construction components and sustainable applications [10]. Additionally, a low-cost gelatin-modified thermo-responsive cement paste was designed, which exhibited enhanced thixotropy through temperature regulation, improved 3D printing performance in cold environments, and maintained or even enhanced post-curing mechanical properties, offering a novel strategy for active rheological control [11]. Furthermore, gelatin has also been used as an air-entraining agent in cementitious materials, significantly increasing mortar porosity (from 0.53% to 2.69%) via reduced air-liquid interfacial tension and Ca^2+^ chelation, while exerting minimal impact on workability and mechanical properties [12]. These advances highlight functionalized gelatin as a novel pathway for the sustainable and high-performance development of cementitious composites.

To further enhance the mechanical properties, crack resistance, and impact resistance of materials, the incorporation of reinforcing fibers has become a widely adopted and effective strategy [13,14]. Studies have shown that various types of natural and synthetic fibers can create bridging effects within cementitious systems, thereby delaying crack propagation, improving toughness, and increasing energy absorption capacity [15,16,17]. Sridhar et al. [18] systematically investigated the effects of pretreated bamboo and jute fibers (with dosages ranging from 0.5% to 2.5% by cement weight) on the compressive and flexural strength of concrete, and identified optimal dosages of 1.5% and 2%, respectively, with good interfacial bonding. Similarly, Zhao et al. [19] confirmed the reinforcing potential of bamboo fibers in ultra-high-performance concrete (UHPC). In contrast, although synthetic polymer fibers are generally more expensive, they offer superior interfacial properties and tensile strength. When incorporated at volume fractions below 2%, they can effectively enhance strain-hardening behavior and promote multiple cracking patterns in engineered cementitious composites (ECC) by optimizing the fiber–matrix interface, thereby exhibiting significant potential for applications under impact, blast, and seismic loading conditions [20,21,22].

From a molecular perspective, amino acids (AAs), the fundamental building blocks of gelatin peptide chains, possess diverse functional groups such as carboxyl (–COOH) and amino (–NH_2_), which enable multipoint coordination with metal ions to form stable chelation networks [23,24]. Such coordination chemistry is ubiquitous in biological systems, serving as a key mechanism in enzymatic catalysis, signal transduction, and nutrient transport. It also provides a theoretical foundation for the design of bioactive molecules and functional nanomaterials. Different metal ions play distinct biological roles: Ca^2+^ promotes bone formation [25], Cu^2+^ enhances angiogenesis at low concentrations [26], and Ag^+^ and Zn^2+^ exhibit strong antibacterial properties [27,28]. Particularly, Mg^2+^ and Fe^3+^ have been approved by the FDA for bone repair applications, underscoring their biocompatibility and clinical relevance [29,30]. Gelatin molecules, rich in carboxyl, amino, and imidazole groups [31], offer abundant coordination sites for these ions, enabling the rapid formation of three-dimensional cross-linked networks under mild conditions. Despite these advances, the synergistic potential of Mg^2+^–gelatin coordination and fiber reinforcement in simultaneously improving mechanical performance, bioactivity, and degradability has not been systematically investigated. Addressing this gap could open new avenues for the design of sustainable bio-based fiber-reinforced composites with multifunctional properties.

Magnesium oxide (MgO), as a source of Mg^2+^, exhibits high reactivity and requires a calcination temperature of only approximately 800 °C, which is significantly lower than that of conventional Portland cement (approximately 1450 °C). Moreover, MgO can achieve complete carbonation during curing, enabling CO_2_ sequestration and contributing to carbon neutrality, thereby effectively reducing both carbon footprint and energy consumption [32]. Therefore, the synergistic integration of gelatin and MgO to construct a coordination-based green composite binder system, in combination with structural optimization via reinforcing fibers, holds great potential for the development of functional construction materials with enhanced strength, impact resistance, and thermal insulation properties.

In this study, bio-gelatin fiber-reinforced composites (BFRCs) were first developed using industrial gelatin and magnesium oxide as primary matrices, combined with various reinforcing fibers. The mechanical properties, impact resistance, and thermal performance were systematically evaluated to elucidate the synergistic reinforcement mechanisms among components. Particular attention was given to the role of different fiber types in enhancing strength, toughness, energy absorption, and thermal behavior. Advanced characterization techniques, including Fourier transform infrared spectroscopy (FTIR) and X-ray diffraction (XRD), were employed to investigate gelatin–Mg^2+^ coordination and its influence on microstructural integrity. In addition, a multi-criteria evaluation using the Technique for Order of Preference by Similarity to Ideal Solution (TOPSIS) was applied to determine the optimal composite formulation. Overall, this work provides theoretical insights and practical guidance for advancing high-performance, eco-friendly composites in sustainable construction applications.

## 2. Experimental Programs

### 2.1. Raw Materials

Industrial gelatin was obtained from Gao Yuan Chemical Co., Ltd. (Changzhou, China). Magnesium oxide (MgO ≥ 99%) was purchased from Zhi Yuan Chemical Reagent Co., Ltd. (Tianjin, China). Quartz sand (50–65 mesh) was obtained from Jing Gong Building Materials Co., Ltd. (Jiangsu, Yancheng, China). Three types of fibers were cut to lengths of 12–15 mm, and their detailed properties are listed in Table 1. Deionized water was used in all experiments.

### 2.2. Fabrication of BFRC

**Figure 1:**
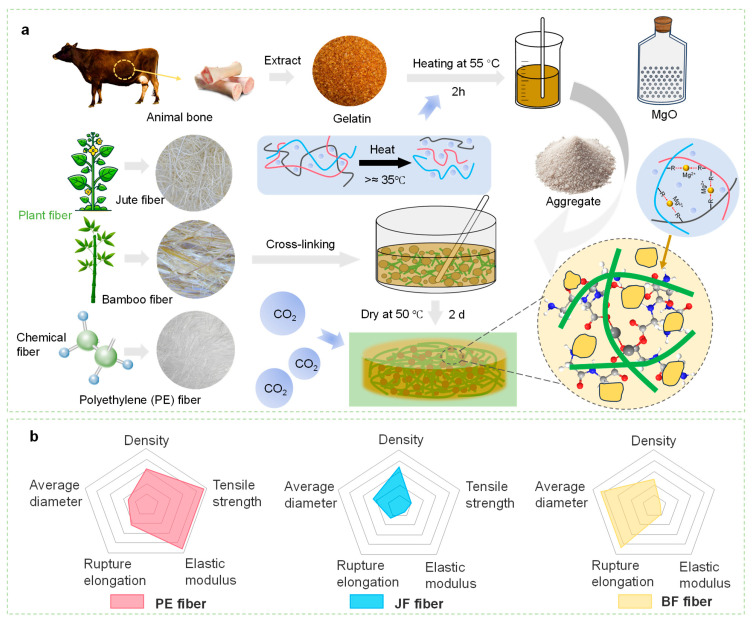
(**a**) Fabrication process of BFRC; (**b**) Fundamental mechanical properties of different fibers (PE, JF, BF).

This study proposes a novel approach using bio-gelatin extracted from animal bones or food waste as a binder, providing a sustainable alternative to traditional cement (Figure 1a). When immersed in water and heated, the hydrogen bonds in gelatin molecules are disrupted, leading to increased fluidity [33]. Subsequently, MgO powder and quartz sand were added into the gelatin solution and mixed at a speed of 140 r/min for 2 min. Afterwards, short-cut fibers (12–15 mm), such as jute (JF) and bamboo (BF), along with synthetic fibers such as polyethylene (PE), were gradually introduced to reinforce the matrix. Once all fibers had been incorporated, the mixture was stirred rapidly at 420 r/min for 2 min until the fibers were fully and uniformly dispersed within the slurry. The composite is then dehydrated and cured at 50 °C, during which Mg^2+^ ions coordinate with gelatin peptide chains [34], forming complexes that improve both strength and stability. Furthermore, magnesium hydroxide is used to absorb CO_2_ and convert it into magnesium carbonate, providing a potential pathway for carbon capture. Figure 1b summarizes the key mechanical properties of PE, JF, and BF fibers, including density, average diameter, tensile strength, elongation at break, and elastic modulus. Comparative analysis shows that the distinct mechanical properties of each fiber influence their reinforcing efficiency in the composite, while Table 2 lists the mix ratios of all raw materials. The fiber content was fixed at 1.5 vol% in this study, ensuring both uniform fiber distribution and effective mechanical enhancement.

### 2.3. Mechanical Properties Testing Procedure

**Figure 2:**
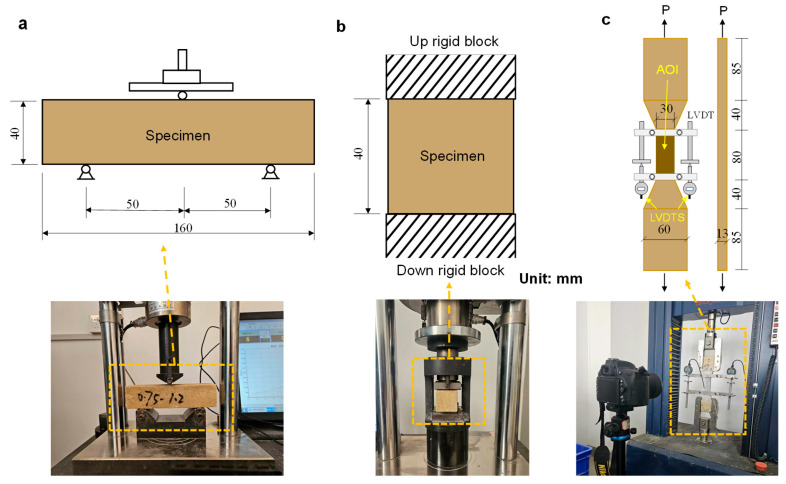
Mechanical performance testing. (**a**) Flexural strength test setup; (**b**) Compressive strength test setup; (**c**) Uniaxial tensile test configuration with LVDT and DIC monitoring.

Compression and flexural strength tests were carried out using a cement mortar testing machine (Dongyi Manufacturing Technology Co., Ltd., Wuxi, China). Tests were performed in accordance with Chinese Standard GB/T 17671-2021 [35]. Flexural specimens (40 × 40 × 160 mm) were loaded at a constant rate of 50 N/s until failure (Figure 2a). Flexural strength was calculated as the average of three peak values. After the flexural test, two halves of each specimen were used for compressive strength testing (Figure 2b). Compressive loading was applied at a constant rate of 2400 N/s until failure. The average compressive strength was obtained from three repeated tests. Tensile tests were performed on dog-bone-shaped specimens using a universal testing machine (Dongyi Manufacturing Technology Co., Ltd., Wuxi, China), following the JSCE standard [36]. A displacement-controlled loading rate of 0.5 mm/min was applied. Deformation within the 80 mm gauge length was measured using two linear variable differential transformers (LVDTs), while digital image correlation (DIC) was used to monitor crack evolution during tension (Figure 2c).

### 2.4. Low-Velocity Impact Test of BFRC

**Figure 3:**
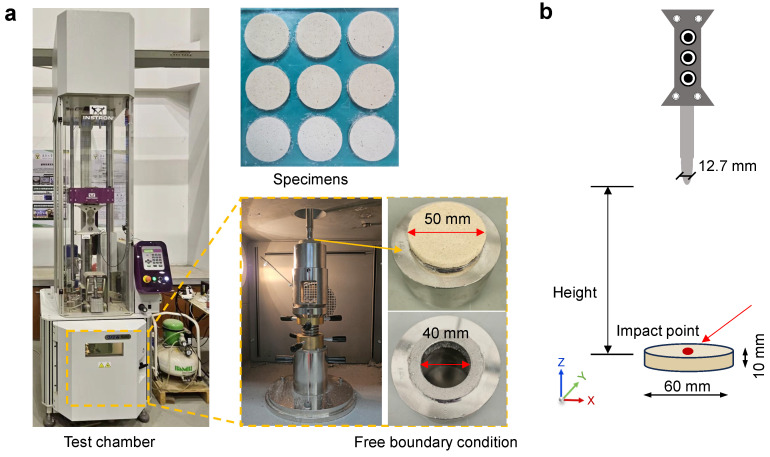
Impact performance testing. (**a**) Schematic diagram of drop-weight impact test and boundary condition setup; (**b**) Dimensions of the impact hammer and specimens.

For the impact test, different BFRC specimens were first conditioned in a thermostatic chamber. A steel hammer with a mass of 2.41 kg was then dropped from three designated heights (50 mm, 100 mm, and 150 mm), corresponding to impact velocities of approximately 0.99, 1.40, and 1.72 m/s, respectively. For each impact velocity, three parallel specimens (*n* = 3) were tested. During the impact process, the drop-weight impact testing machine (Type CEAST-9350, Instron Co., Ltd. Norwood, MA, USA) recorded the impact force and displacement time history through a force sensor integrated into the data acquisition system (Figure 3). The data acquisition frequency was set to 20 kHz. The impact force ($F_{t}$, N), velocity ($v_{t}$, m/s), and displacement ($\delta_{t}$, *m*) were automatically recorded by sensors embedded in the impactor. The absorbed energy ($E_{t}$, *J*) was calculated based on the ASTM D7136 [37], following the computational procedure outlined below.

(1) $$v_{t}={\;v}_{0}+gt-\int_{0}^{t}\frac{F\left(\tau \right)}{m}d\tau$$

(2) $$\delta_{t}=\delta_{0}+v_{0}t+\frac{gt^{2}}{2}-\int_{0}^{t}\int_{0}^{t}\frac{F\left(\tau \right)}{m}d^{2}\tau$$

(3) $$E_{t}=\frac{m\left[v_{0}^{2}-v^{2}\left(t\right)\right]}{2}+mg\delta \left(t\right)$$

where $v_{0}$ and $\delta_{0}$ represent the initial velocity and position, respectively, *g* is the gravitational acceleration, and *m* denote the applied mass.

### 2.5. Thermal Performance Measurement

**Figure 4:**
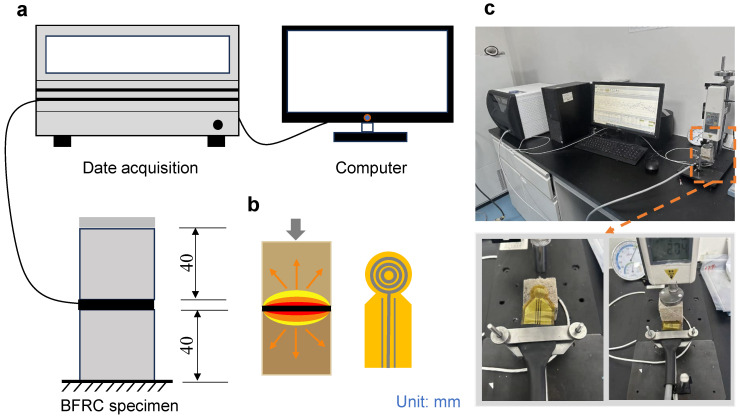
Thermal performance testing setup. (**a**) Schematic of TPS 2500 S thermal constants analyzer measurement setup; (**b**) Structure and design of the spiral double-wire thermal disk sensor; (**c**) Experimental setup for thermal performance measurement.

The thermal properties of BFRC were evaluated using the Transient Plane Source (TPS) method [38]. Measurements were conducted using a TPS 2500 S thermal constants analyzer (Hot Disk AB, Göteborg, Sweden), as shown in Figure 4a. The Kapton-insulated sensor, designed as a spiral bifilar thermal disk (diameter 3.189 mm), functioned simultaneously as a resistive heater and a resistance thermometer [39]. Internally, it consists of multiple concentric annular heating elements (Figure 4b). For testing, two cubic BFRC specimens (40 × 40 × 40 mm^3^) were prepared, and the sensor was symmetrically placed at their interface (Figure 4c). A metal weight was applied on top of the assembly to ensure good thermal contact and stable measurements by maintaining constant pressure. This setup minimized interfacial thermal resistance and ensured repeatable, accurate thermal conductivity measurements.

Thermal characterization was performed by applying a constant electrical current to the sensor, inducing resistive heating in the spiral element. The generated heat diffused radially into the surrounding material, while the sensor’s temperature-dependent resistivity enabled real-time monitoring of thermal response. Resistance variations were recorded at 200 time intervals during heating, enabling determination of temperature evolution over time. The data were processed to derive the thermal conductivity, thermal diffusivity, and volumetric heat capacity of BFRC. To ensure purely conductive heat transfer and eliminate convection, all measurements were conducted in a nitrogen-purged environment. The variation in sensor resistance, serving as the primary output signal, follows the theoretical relationship [40]:

(4) $$R\left(t\right)=R_{0}\left[1+\Omega \left[\Delta T_{i}+\Delta T_{ave}\left(\tau \right)\right]\right]$$

where $R_{0}$ denotes the initial resistance of the thermal disk at *t* = 0, Ω represents the temperature coefficient of resistivity, $\Delta T_{i}$ indicates a constant temperature offset, and $T_{ave}(\tau )$ corresponds to the average temperature rise on the opposite side of the Kapton insulation as a function of time τ.

### 2.6. Infrared Spectroscopy Testing

Fourier transform infrared spectroscopy (FTIR) analysis was performed using a Nicolet iS10 spectrometer (Thermo Fisher Scientific, Waltham, MA, USA) within a spectral range of 400~4000 cm^−1^. Prior to testing, the specimen was finely ground to pass through a 200-mesh sieve. The powdered sample was then thoroughly mixed with potassium bromide (KBr), placed into a mold, and compressed into pellets. Finally, the prepared sample was mounted on the sample holder and positioned in the test chamber for spectral analysis.

### 2.7. X-Ray Diffraction (XRD) Characterization

X-ray diffraction (XRD) measurements were performed using a D8 ADVANCE diffractometer (Bruker, Ettlingen, Germany). The instrument was operated at an accelerating voltage of 40 kV and a tube current of 40 mA, employing a Cu *Kα* radiation source with a wavelength of λ = 1.5406 Å. Data were collected in step-scan mode over a 2θ range of 5° to 90°, with a scanning rate of 10°/min. The total measurement time was controlled within 10 min.

### 2.8. Scanning Electron Microscopy (SEM)

To investigate the microstructure of the different fragment series, a Gemini300 field-emission scanning electron microscope (FE-SEM) (Carl Zeiss AG, Jena, Germany) was utilized. Prior to observation, the specimens underwent an initial drying process. A thin conductive layer was then deposited using a high-resolution sputter coater, with platinum serving as the target material and a coating duration of 360 s. Secondary electron mode was used for imaging, operating at an accelerating voltage of 15 kV and an approximate working distance of 8 mm.

## 3. Results

### 3.1. Mechanical Properties Test Results

**Figure 5:**
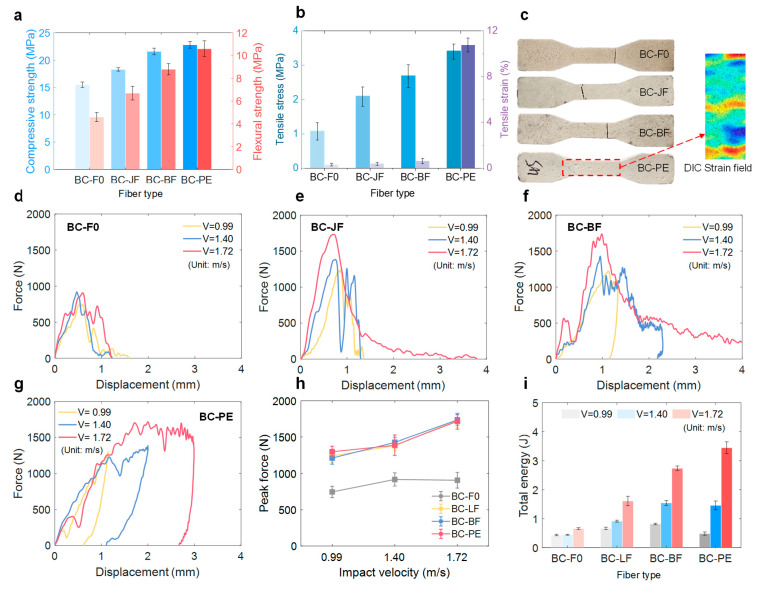
Mechanical performance of BFRCs with different fiber types. (**a**) Compressive and flexural strengths; (**b**) Tensile stress and strain; (**c**) Tensile failure patterns; (**d**–**g**) Impact responses at different velocities; (**h**) Peak impact force; (**i**) Energy absorption under various impact velocities.

Compressive and flexural strength tests revealed that fiber type significantly influenced the mechanical properties of BFRC (Figure 5a). The plain matrix specimen (BC-F0) showed the lowest compressive (15.5 MPa) and flexural strength (4.6 MPa). Adding jute fiber (BC-JF) produced slight improvements in both strengths, indicating a limited reinforcing effect. This was attributed to the inherently low stiffness and strength of jute fibers, which limit stress distribution and load-bearing capacity. In contrast, the BC-BF specimen, reinforced with BF, showed a marked improvement in performance, with compressive strength reaching 21.6 MPa and flexural strength increasing to 8.8 MPa [41]. The superior strength and rigidity of bamboo fibers enable effective bridging under compressive and bending loads, thereby enhancing the composite’s overall mechanical behavior [42]. The best performance was achieved by PE fiber reinforcement (BC-PE), with compressive and flexural strengths of 22.8 and 10.6 MPa, respectively. This improvement was attributed to the excellent mechanical properties of PE fibers and their effective interfacial bonding with the matrix [20,43].

Figure 5b compares the tensile stress–strain responses of specimens reinforced with different fiber types. All fiber-reinforced composites exhibited markedly higher tensile strength and ductility compared with the fiber-free BC-F0 (average tensile strength: 1.07 MPa; tensile strain: 0.3%). Specifically, BC-JF achieved an average tensile strength of 2.09 MPa and a strain of 0.39%, representing improvements of 102% and 9%, respectively. BC-BF reached 2.68 MPa and 0.62%, corresponding to increases of 150% in strength and 106.7% in strain. The BC-PE group showed the most remarkable enhancement, with tensile strength rising to 3.40 MPa and strain soaring to 10.77%. This exceptional performance is mainly attributed to the superior mechanical properties of PE fibers and their effective crack-bridging capability [44]. Figure 5c presents the fracture patterns of the tensile specimens. The BC-F0, BC-JF, and BC-BF groups mainly exhibited single central cracks. In contrast, the BC-PE specimens showed not only a major crack but also a large number of fine microcracks distributed throughout the tensile zone. DIC analysis further confirmed the microcrack distribution in this region, which contributed to improved tensile strength and ductility [45].

Furthermore, to evaluate the impact resistance of the composites, low-velocity drop-weight impact tests were conducted. Results showed that, compared to BC-F0, all fiber-reinforced specimens exhibited significantly higher peak impact forces across different impact velocities, particularly at *v* = 1.72 m/s. The force–displacement curves (Figure 5d–g) revealed distinct mechanical responses based on fiber type. The BC-JF specimens showed smoother curves with stable displacement at lower velocities but steeper curves under high-velocity impacts, suggesting potential stress concentration and increased brittleness due to JF. The BC-BF specimens showed larger displacements under high impact velocities, accompanied by relatively high peak forces followed by a rapid post-peak drop, indicating mixed ductile-brittle failure. In contrast, the BC-PE specimens exhibited a typical three-stage response: an initial peak force, a strain-hardening plateau, and a gradual post-peak decline [46]. This behavior markedly improved the peak impact force and impact energy absorption (Figure 5h,i).

**Figure 6:**
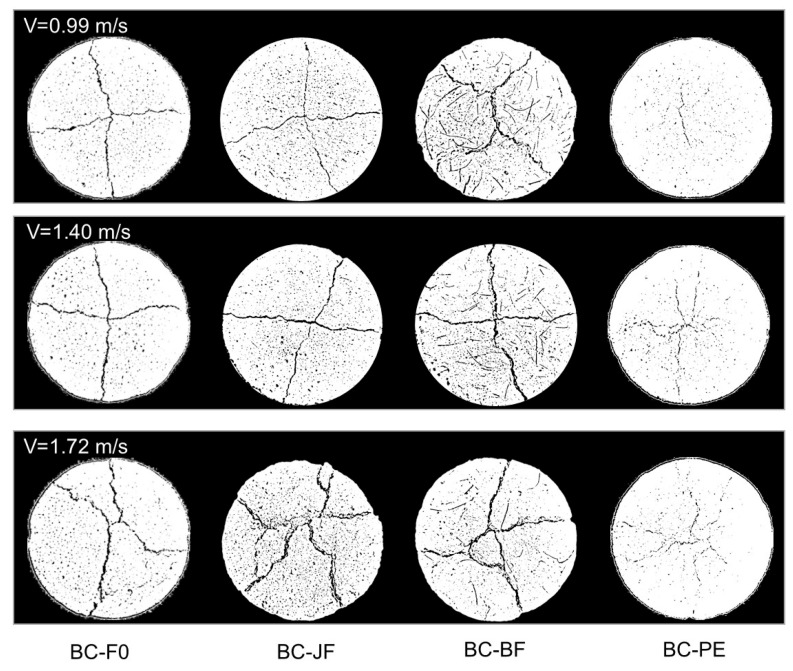
Comparison of failure patterns at different impact velocities.

Figure 6 shows the failure patterns of BFRC (BC-F0, BC-JF, BC-BF, and BC-PE) under three impact velocities (*v* = 0.99, 1.40, and 1.72 m/s). As the impact velocity increased, more extensive crack propagation and greater damage were observed. The BC-F0 specimen already exhibited full-penetration cracks indicative of brittle failure at *v* = 0.99 m/s. Although BC-JF and BC-BF specimens showed some improvement, visible primary cracks still formed. In contrast, the BC-PE group displayed evenly distributed fine cracks without penetration, indicating superior crack resistance. At *v* = 1.40 m/s, crack propagation intensified in BC-F0 and BC-JF, while BC-BF exhibited slightly improved toughness. The BC-PE specimen maintained its fine, web-like crack distribution and avoided significant structural failure. Under the highest impact velocity of 1.72 m/s, severe cracking and extensive damage occurred in all specimens except BC-PE. The BC-PE sample exhibited dense but non-penetrative cracks, confirming its excellent crack control and impact resistance.

### 3.2. Thermal Performance

**Figure 7:**
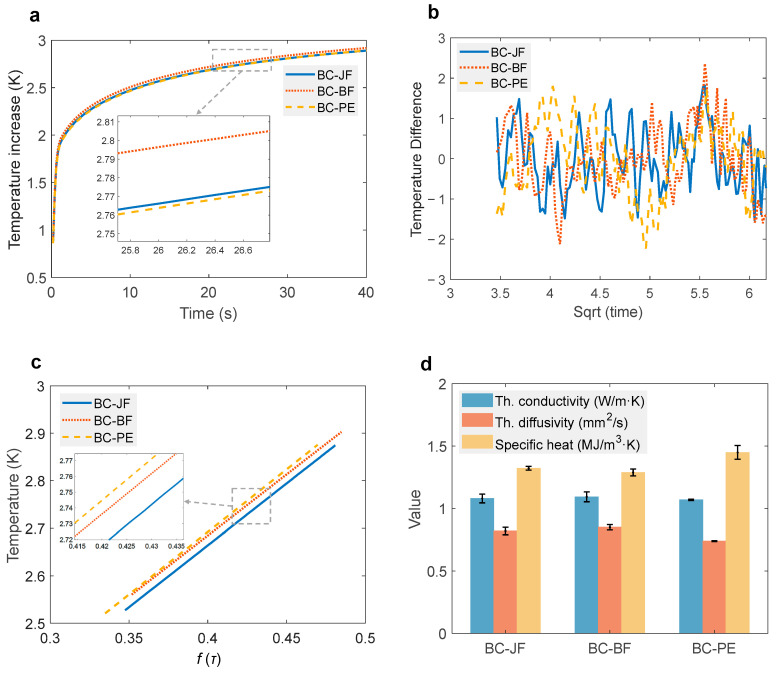

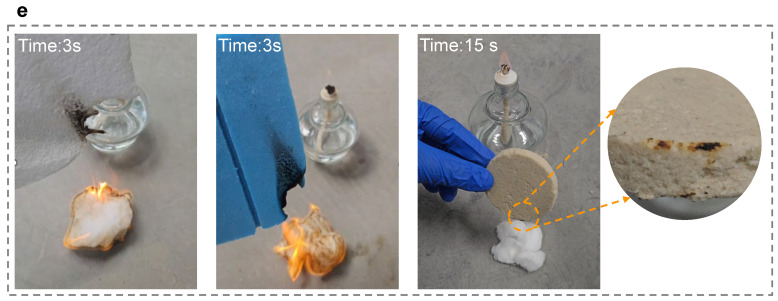
Thermal and fire performance of BFRCs. (**a**) Temperature evolution under constant heat flux; (**b**) Temperature difference versus square root of time; (**c**) Temperature versus dimensionless thermal time function *f*(*τ*); (**d**) Thermophysical properties (thermal conductivity, diffusivity, and specific heat capacity); (**e**) Flame exposure tests comparing BFRCs with conventional polymeric insulation materials (EPS and XPS).

As shown in Figure 7a, all specimens exhibited a comparable trend in temperature evolution under constant heat flux. An initial rapid increase in temperature was observed, followed by a gradual approach to thermal equilibrium. Notably, BC-BF exhibited a marginally faster temperature rise during the first 10 s, which is attributed to its relatively higher thermal diffusivity. Nonetheless, as the temperature approached 3 K, the differences among the materials became negligible, suggesting a convergence in thermal response at steady-state conditions.

To further examine transient thermal behavior, the temperature difference as a function of the square root of time was plotted (Figure 7b). All samples exhibited relatively small fluctuation amplitudes with consistent oscillation trends throughout the testing period. This indicates that, despite their compositional differences, the materials possess comparable thermal responsiveness under transient thermal conditions [47]. In Figure 7c, a near-linear relationship between temperature and the dimensionless thermal time function *f* (*τ*) was observed for all composites. The consistency in slope across different samples implies similar heat conduction mechanisms and confirms the reliability of the experimental measurements.

Thermophysical properties are summarized in Figure 7d and Table 3. The thermal conductivities of the three materials were found to be in a narrow range: 1.080 W·m^−1^·K^−1^ (BC-JF), 1.093 W·m^−1^·K^−1^ (BC-BF), and 1.069 W·m^−1^·K^−1^ (BC-PE), indicating minimal variation in their heat transfer capabilities. However, notable differences were observed in thermal diffusivity and specific heat capacity. BC-BF exhibited the highest thermal diffusivity (0.85 mm^2^·s^−1^), indicative of faster internal heat propagation, while BC-PE demonstrated the highest specific heat capacity (1.4487 MJ·m^−3^·K^−1^), reflecting enhanced heat absorption and storage potential.

As shown in Figure 7e, direct flame exposure tests were conducted to assess fire performance differences between conventional polymeric insulation materials and the tested composites. EPS and XPS panels ignited rapidly within 3 s, showing pronounced melt-dripping behavior and igniting adjacent cotton. XPS, in particular, emitted dense black smoke during combustion, posing significant safety risks. Conversely, the BC-BF composite exhibited superior flame resistance; even after 15 s of continuous flame exposure, no dripping or sustained flaming occurred, and the adjacent cotton remained unignited. This demonstrates its potential as a flame-retardant building material. It should be emphasized that the flame exposure test presented in this work (Figure 7e) was intended only as a preliminary visual demonstration to contrast the ignition behavior of BFRCs with conventional polymeric foams (EPS/XPS). Future studies will employ standardized methods (e.g., cone calorimetry, UL-94 vertical burning test, ISO 834 furnace testing) to comprehensively evaluate the fire behavior of BFRCs under regulatory conditions.

## 4. Discussions

**Figure 8:**
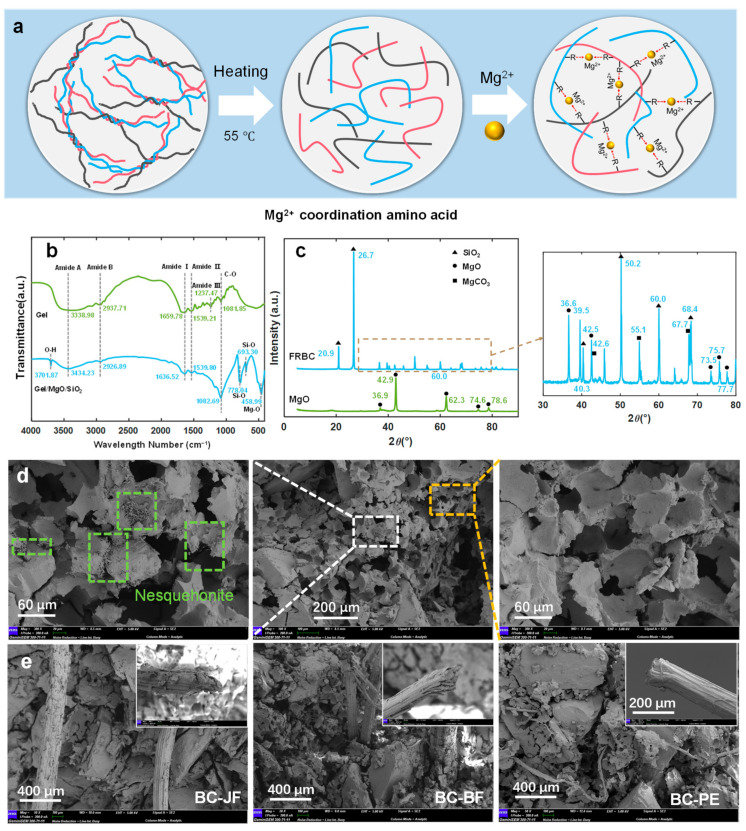
Coordination mechanism and microstructural characterization of BFRCs. (**a**) Schematic of gelatin–Mg^2+^ coordination and triple-helix complex formation. (**b**) FTIR spectra showing characteristic bands, Mg^2+^ coordination, and quartz integration; (**c**) XRD patterns indicating phase composition, partial carbonation, and crystallinity of quartz and MgO; (**d**) SEM image of honeycomb-like porous morphology with nesquehonite crystal formation; (**e**) Fracture morphologies of fiber-reinforced composites after impact, highlighting fiber fracture and pull-out mechanisms.

The collagen proteins in gelatin form a colloidal solution upon dissolution in water. When heated to approximately 55 °C water molecules disrupt some hydrogen bonds, leading to partial unwinding of the collagen polypeptide chains [48]. Subsequently, Mg^2+^ ions coordinate with carboxyl (-COO^−^) and amino (-NH_2_) groups in the gelatin molecules, forming stable triple-helical coordination complexes (Figure 8a).

Figure 8b presents the FTIR analysis of the BFRC. The gelatin matrix exhibits characteristic absorption bands at 3434 cm^−1^ (Amide A, N-H/O-H stretching vibrations), 2938 cm^−1^ (Amide B, asymmetric C-H stretching), 1659 cm^−1^ (Amide I, C=O stretching vibration), and 1539 cm^−1^ (Amide II, coupling of N-H bending and C-N stretching vibrations) [49]. The addition of MgO and quartz sand induced two key spectral changes: First, the Amide I band redshifted by 22.48 cm^−1^ to 1636.52 cm^−1^, indicating Mg^2+^ coordination with amino acid residues in the gelatin molecules. Second, new peaks at 458 cm^−1^ (Mg-O) and 778 cm^−1^ (Si-O-Si) confirmed Mg^2+^-Gel interactions and quartz sand incorporation [24,50]. Notably, the introduced quartz sand not only enhanced interfacial bonding between the Si-O network and organic matrix but also potentially established synergistic effects with the Mg^2+^-Gel coordination network, thereby effectively improving the microstructural densification of BFRC and subsequently enhancing its mechanical properties.

Figure 8c illustrates the phase composition of the BFRC. Distinct diffraction peaks are observed at 2θ = 26.7°, 50.2°, 60.0°, 68.4°, and 75.7°, corresponding to the (101), (112), (211), (203), and (205) planes of α-quartz (SiO_2_, JCPDS 46-1045), respectively. The primary peak at 2θ = 26.7° exhibits a full width at half maximum (FWHM) of 0.12°, and the average crystallite size is calculated to be approximately 45 nm using the Scherrer equation, indicating that the quartz phase retains a high degree of crystallinity [40]. In addition, diffraction peaks at 2θ = 36.6°, 42.5°, and 62.3° are assigned to the (111), (200), and (220) planes of cubic-phase periclase (MgO, JCPDS 75-0447). Compared with the reference sample of pure MgO, the corresponding peak intensities decrease by approximately 40%, which may be attributed to partial amorphization of MgO and its surface coordination interactions with gelatin molecules. Weak diffraction peaks appearing at 2θ = 39.5°, 55.1°, and 67.7° are tentatively assigned to the (104), (113), and (116) planes of magnesite-type MgCO_3_ (JCPDS 08-0479), indicating that partial carbonation of MgO may have occurred under environmental conditions [51]. Notably, a broad diffraction feature in the range of 2θ ≈ 20~25° (corresponding to a d-spacing of approximately 4.2 Å) suggests the presence of a partially ordered structure. This structural feature is likely related to the coordination between Mg^2+^ ions and gelatin molecules. The coordination effect is further corroborated by FTIR analysis, in which the amide I band exhibits a redshift of approximately 22 cm^−1^, supporting the formation of such metal-biopolymer interactions.

Figure 8d exhibits a typical honeycomb-like porous morphology of BFRC. This structure facilitates the dispersion of external impact stresses and enhances energy absorption capacity, thereby significantly improving its impact resistance. Additionally, the entrapped air within the porous framework effectively reduces heat transfer, resulting in low thermal conductivity. Moreover, a large number of needle-like crystalline structures can be observed attached to the pore walls. These structures are likely nesquehonite (MgCO_3_·3H_2_O) crystals, formed under alkaline or hydrothermal conditions via the reaction of MgO with CO_2_ from the environment [52]. The presence of these crystals not only reinforces the microstructural stability but may also enhance the interfacial bonding between the inorganic and organic phases. Figure 8e further presents the cross-sectional morphologies of three different fiber-reinforced BFRCs after impact failure. Distinct necking phenomena are observed in BC-JF and BC-BF samples, indicating tensile fracture of the fibers under impact loading. In contrast, the BC-PE specimen predominantly exhibits fiber pulled-out behavior, with fibers remaining largely intact and showing no apparent fracture surfaces [53]. Compared with the tensile fracture mechanism observed in natural fibers, PE fibers demonstrate superior tensile strength and interfacial bonding capacity, which facilitate pulled-out and slippage under impact loads. This mechanism effectively enhances the bridging effect between the fibers and the matrix, thereby improving the impact strength and toughness of the BFRC.

## 5. Multi-Criteria Performance Evaluation

To comprehensively evaluate the performance of four BFRC materials across multiple dimensions, including mechanical properties, impact resistance, and thermal conductivity, the Technique for Order Preference by Similarity to an Ideal Solution (TOPSIS) model was employed for comparative analysis [45]. The evaluation model incorporates six performance indicators: compressive strength, flexural strength, uniaxial tensile strength, uniaxial tensile strain, impact energy absorption, and thermal conductivity. Based on performance objectives, these indicators can be categorized into two types: the first five are benefit-type indicators (i.e., higher values are preferable), while thermal conductivity is a cost-type indicator (i.e., lower values are preferable). The latter requires forward normalization using the following formula:

(5) $$x_{i}^{\prime }=\frac{1}{x_{i}}$$

where $x_{i}^{\prime }$ represents the normalized value, $x_{i}$ is the original value. Subsequently, an evaluation matrix X was constructed for BFRC (BC-0, BC-JF, BC-BF, BC-PE):

(6) $$X=\left[\begin{array}{ccc} x_{11} & \ldots & x_{16}\\ \vdots & \ddots & \vdots \\ x_{41} & \ldots & x_{46} \end{array}\right]\left(i=1,2,\ldots ,4;\;j=1,2,\ldots ,6\right)$$

where $x_{ij}$ represents the original test value of the *i*-th material under the *j*-th performance indicator. The normalized matrix was then processed to eliminate dimensional effects using vector normalization:

(7) $$z_{ij}=\frac{x_{ij}}{\sqrt{\sum_{i=1}^{n}x_{ij}^{2}}}$$

After normalization, the positive ideal solution ($Z^{+}$) and the negative ideal solution ($Z^{-}$) for each indicator were determined as follows:

(8) $$\left\{\begin{array}{c} Z^{+}=\max z_{1j},\;\max z_{2j},\ldots ,\;\max z_{6j}\\ Z^{-}=\min z_{1j},\;\min z_{2j},\ldots ,\;\min z_{6j} \end{array}\right.$$

Next, the weighted distances from each specimen to the positive and negative ideal solutions were calculated:

(9) $$D_{i}^{-}=\sqrt{\sum_{j=1}^{n}\omega_{j}{\left(Z_{j}^{-}-z_{ij}\right)}^{2}};\;D_{i}^{-}=\sqrt{\sum_{j=1}^{n}\omega_{j}{\left(Z_{j}^{-}-z_{ij}\right)}^{2}}$$

where $\omega_{j}$ denotes the weight of the *j*-th indicator. Equal weights were assigned (i.e., $\omega_{j}$ = 1/6). Finally, the comprehensive evaluation score ($S_{i}$) for each sample was computed:

(10) $$S_{i}=\frac{D_{i}^{-}}{D_{i}^{+}+D_{i}^{-}}$$

A higher $S_{i}$ value indicates that the material is closer to the ideal solution, signifying superior overall performance.

**Figure 9:**
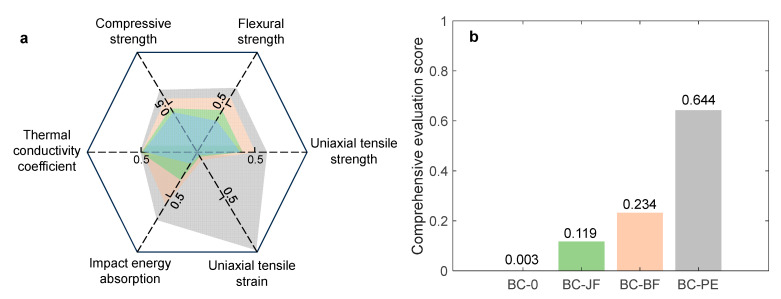
Comprehensive performance evaluation of BFRCs using the TOPSIS model. (**a**) Radar chart of six normalized performance indicators for each composite; (**b**) Overall TOPSIS scores highlighting the superior performance of BC-PE and the reinforcing effects of different fiber types.

The comprehensive performance evaluation results of the four BFRC materials based on the TOPSIS model are presented in Figure 9. Figure 9a shows a radar chart constructed from six normalized indicators, visually illustrating the relative performance of each material across multiple dimensions. The results indicate that BC-PE exhibits superior performance in most indicators, particularly in tensile properties and impact energy absorption. In contrast, BC-0 demonstrates the lowest performance across all dimensions, highlighting the positive reinforcing effect of fiber incorporation on material properties. Figure 9b presents the bar chart of the comprehensive evaluation scores calculated using the TOPSIS method. BC-PE achieves the highest score (0.644), indicating that its overall performance is closest to the ideal solution. BC-BF and BC-JF show intermediate performance with scores of 0.234 and 0.119, respectively. BC-0 has the lowest score (0.003), suggesting the weakest overall performance. In summary, BC-PE demonstrates significant advantages in both mechanical and thermal functionalities, indicating its strong potential for application in high-performance composite materials.

## 6. Conclusions

In this study, bio-gelatin fiber-reinforced composites (BFRCs) were fabricated by utilizing industrial gelatin and magnesium oxide as the main matrix materials, reinforced with various types of fibers. The mechanical, impact, and thermal properties of the composites were systematically investigated, and the underlying reinforcement mechanisms were clarified. The main conclusions are as follows:

(1) Fiber incorporation markedly enhanced BFRC performance. Bamboo fiber (BC-BF) improved compressive and flexural strength, whereas PE fiber (BC-PE) showed the best overall results, with compressive, flexural, and tensile strengths of 22.8, 10.6, and 3.40 MPa, respectively, and a tensile strain of 10.77%. The superior crack-bridging ability of PE fibers promoted multiple microcracks, improving toughness and load capacity. Impact tests confirmed that BC-PE exhibited the highest peak impact force and energy absorption, outperforming all other fiber types.
(2) Thermal conductivities of fiber-reinforced composites were comparable, but thermal diffusivity and specific heat capacity differed. BC-BF showed higher thermal conductivity, while BC-PE demonstrated superior heat storage and buffering capacity. Flame exposure tests further confirmed the excellent flame-retardant behavior of BFRCs, indicating favorable thermal safety.
(3) Microstructural analysis showed that gelatin formed a stable triple-helix structure upon heating and coordination with Mg2+, enhancing matrix compactness and integrity. FTIR and XRD confirmed gelatin–Mg2+ coordination and the synergistic effect of quartz sand, which together improved thermal stability and strength. A porous honeycomb structure facilitated stress distribution and energy dissipation, while the fiber pull-out mechanism of PE under impact provided strong interfacial bonding and toughening effects.
(4) Multi-criteria evaluation using the TOPSIS model identified BC-PE as the best-performing composite, with optimal tensile properties, impact resistance, and thermal regulation. This work provides insights and theoretical support for advancing bio-based composites in impact-resistant and functional insulation applications.

In addition to the laboratory-scale results, future work will focus on field demonstrations in real construction and packaging applications, in order to assess the durability, environmental stability, and cost-effectiveness of BFRCs under practical service conditions.

## Figures and Tables

**Table 1:** Physical and mechanical properties of fibers.

Fiber Type	Length (mm)	Diameter (μm)	Density (g/cm^3^)	Tensile Strength (MPa)	Elastic Modulus (GPa)	Rupture Elongation (%)
Jute fiber	12~15	60	1.4	400	10	0.5
Bamboo fiber	12~15	120	1.1	350	22	5.8
PE fiber	12	24	0.97	3000	110	2.7

**Table 2:** Mix proportion of BFRC.

Mix ID	By Weight (kg/m^3^)				By Volume (%)		
	Gelatin	Water	QS	MgO	JF	BF	PE
BC-0	154.6	120.5	1125	35	0	0	0
BC-LF	154.6	120.5	1125	35	1.5	0	0
BC-BF	154.6	120.5	1125	35	0	1.5	0
BC-PE	154.6	120.5	1125	35	0	0	1.5

**Table 3:** Thermal properties test results.

Specimens	Temperature (°C)	Ave. Th. Conductivity (W/mK)	Ave. Th. Diffusivity (mm^2^/s)	Ave. Specific Heat (MJ/m^3^K)
BC-JF	22.8 °C	1.080	0.82	1.3215
BC-BF	22.8 °C	1.093	0.85	1.2879
BC-PE	23.0 °C	1.069	0.74	1.4487

## Data Availability

The original contributions presented in this study are included in the article. Further inquiries can be directed to the corresponding authors.
